# Polymer-Based Thermal Protective Composites: The Role of Reinforcement and Matrix in Providing Strength and Fire Resistance

**DOI:** 10.3390/polym17101419

**Published:** 2025-05-21

**Authors:** Mohammed Meiirbekov, Assem Kuandyk, Mukhammed Sadykov, Meiir Nurzhanov, Nurmakhan Yesbolov, Berdiyar Baiserikov, Ilyas Ablakatov, Laura Mustafa, Botagoz Medyanova, Arman Kulbekov, Sunkar Orazbek, Abussaid Yermekov

**Affiliations:** 1 JSC “National Center of Space Research and Technology”, Almaty 050010, Kazakhstan; m.sadykov@spaceres.kz (M.S.); m.nurzhanov@spaceres.kz (M.N.); n.yesbolov@spaceres.kz (N.Y.); b.baiserikov@spaceres.kz (B.B.); ablakatov.i@spaceres.kz (I.A.); l.mustafa@spaceres.kz (L.M.); medyanova.b@spaceres.kz (B.M.); a.kulbekov@spaceres.kz (A.K.); a.yermekov@spaceres.kz (A.Y.); 2 Faculty of Mechanics and Mathematics, Al-Farabi Kazakh National University, Almaty 050040, Kazakhstan; 3 School of Materials Science and Green Technologies, Kazakh-British Technical University, Almaty 050005, Kazakhstan; 4 Mining and Metallurgical Institute Named After O.A. Baikonurov, Kazakh National Research Technical University Named After K.I. Satbayev, Almaty 050043, Kazakhstan; 010722501137-d@stud.satbayev.university

**Keywords:** thermal-resistant composites, fabric reinforcement, phenol-formaldehyde resin, epoxy resin, mechanical properties, fire resistance, thermal conductivity

## Abstract

This study addresses the need for thermomechanically robust materials for high-temperature environments by investigating fabric-reinforced composites produced through polymer infiltration and thermal pressing using phenol-formaldehyde (PF) and epoxy (ER) resins. Experimental validation was required due to the lack of comparative data across different textile reinforcements under identical conditions. Seven technical fabrics—carbon, aramid, basalt, silica, fiberglass, asbestos, and a carbon/aramid hybrid—were used as reinforcements. Mechanical testing revealed that carbon- and hybrid fiber composites exhibited the highest tensile (up to 465 MPa) and compressive strengths (up to 301 MPa), particularly when combined with ER. Conversely, the use of PF generally resulted in a 30–50% reduction in mechanical strength. However, PF-based composites demonstrated superior thermal resistance, with the silica/PF combination showing the lowest back-face temperature (401 °C), up to 37% lower than other pairings. Thermal conductivity ranged from 0.041 to 0.51 W/m·K, with PF-based systems offering 6–12% lower values on average compared to ER-based analogs. Morphological analysis confirmed better interfacial bonding in ER composites, while PF systems showed higher structural integrity under thermal loading. Overall, the results emphasize the trade-offs between mechanical strength and thermal protection depending on the fabric–resin combination. Among all variants, the silica fabric with PF demonstrated the most balanced performance, making it a promising candidate for thermomechanical applications.

## 1. Introduction

Modern rocket systems impose stringent requirements on thermal protection systems (TPSs), which must operate under intense heat fluxes and significant mechanical loads. These requirements are driven by the extreme conditions encountered in rocket engines, where temperature and pressure can fluctuate dramatically, generating unique stresses for protective materials [1,2]. In solid rocket motors (SRMs), temperature gradients in the combustion chamber can exceed 3000 °C, leading to rapid thermal degradation and the reduced service life of structural components. This necessitates the development of materials capable of withstanding such high temperatures, ensuring the durability and reliability of systems under demanding operational conditions [3].

In this context, the development of composite thermal insulation materials with tailored thermomechanical properties is of particular relevance. The selection of both reinforcing and matrix components, as well as the structural formation technique, plays a decisive role in the performance of these materials [4].

Reinforcing fibers are among the most critical components in these materials. They significantly influence the strength, thermal resistance, and oxidative stability of the composite. Commonly used types of reinforcement include carbon, aramid, basalt, silica, fiberglass, and others [5,6,7,8,9,10,11,12,13]. Each fiber type brings distinct characteristics that determine the final performance of the material.

Carbon fibers, for instance, exhibit excellent strength and thermal stability, making them ideal for use under extreme temperatures. They offer high specific strength (up to 5 GPa), low thermal conductivity (~0.2–0.4 W/m·K), and thermal resistance up to 2500 °C in inert atmospheres [5,6,7]. Basalt fibers provide thermal stability up to 900 °C, as well as resistance to thermal shock and alkaline corrosion, making them suitable for chemically aggressive environments [8,9]. Silica fibers are known for their high chemical inertness and temperature resistance up to 1000 °C [10], while aramid fibers contribute to strength under dynamic and impact loads [11]. Glass fabric composites can retain considerable post-impact strength, especially with surface-modified fibers [12]. Asbestos fibers, despite their high thermal resistance, are limited in use due to their propensity for degradation and the formation of hazardous by-products under heat [13].

However, reinforcing fibers alone cannot ensure the mechanical integrity and stability of the composite at elevated temperatures. The matrix materials, which bind the fibers and maintain structural cohesion, are equally vital. Among the most widely used matrix systems are phenol-formaldehyde (PF) and epoxy (ER) resins, both of which play a crucial role in tailoring the final properties of the composite.

In recent years, a number of studies have been published focusing on thermal protection composites based on PF and ER. These works cover both experimental and numerical approaches to investigating thermal resistance, ablation behavior, matrix degradation, and mechanical performance under thermo-oxidative loading [14,15,16,17,18]. In particular, the analysis of the recent literature has shown that modified PF resins demonstrate stability and high thermal resistance under ablation conditions [3,19,20,21,22,23]. Studies have also addressed ER-based composites used in aerospace thermal protection coatings, with an emphasis on binder structure and fiber–matrix interactions [24,25,26,27,28].

PF resins are renowned for their high flame resistance and ablation durability, though their mechanical strength and thermal endurance are somewhat limited (typically up to ~250 °C) [20,21,23]. These resins are suitable for applications requiring high thermal stability at moderate operating temperatures. In contrast, ERs offer significantly higher mechanical strength (up to 90 MPa in tensile applications) and excellent fiber wetting, which is critical for composite integrity [27,29,30]. Epoxies are particularly effective in highly loaded structural applications where both mechanical robustness and environmental resistance are essential.

Thus, the choice of fiber and matrix components directly influences the operational capabilities of the resulting composite. Finding an optimal combination is essential to meet the requirements for strength, heat resistance, and oxidative stability under extreme loads.

Composite systems employing similar fiber–matrix combinations have been successfully applied in aerospace engineering. Phenolic–carbon composites used in Space Shuttle and SLS Boosters provide ablative protection through controlled material recession. The Ariane-5 launcher incorporates ablative composites with ceramic inserts, reducing thermal conductivity by 30–40% compared to monolithic materials. In systems like the SLS Booster, metal–ceramic coatings based on aluminum and zirconium reflect up to 60% of infrared radiation [2,31,32].

A critical aspect of composite manufacturing is the structural formation method. Common approaches include sintering, fiber infiltration, polymer molding, and additive manufacturing [33,34,35,36]. In the present study, a polymer infiltration and thermal pressing method was selected. This approach enables the production of materials with optimized density, controlled porosity, and uniform matrix distribution, which are crucial for performance under combined thermal and mechanical loads [34,37,38,39].

The objective of this study is to investigate the manufacturing process for high-temperature thermal insulation composites based on polymer infiltration with pressing. Seven types of reinforcement fabrics and two types of matrix resins (PF and ER) were used. The resulting composites were subjected to a comprehensive set of thermal and mechanical tests.

## 2. Materials and Methods

### 2.1. Materials

In this study, various technical fabrics were used as reinforcing fillers: carbon fabric (JSC “UMATEX”, Moscow, Russia), silica fabric (JSC “NPO Stekloplastik named after N.N. Trofimov”, Solnechnogorsk, Russia), aramid fabric (LLC “Aramid”, Kamensk-Shakhtinsky, Russia), basalt fabric (“Polotsk-Steklovolokno”, Polotsk, Belarus), fiberglass fabric (“Armplast”, Nizhny Novgorod, Russia), hybrid carbon/aramid fabric (Jiangsu Huaiying New Material Co., Ltd., Xinyi, China), and asbestos fabric (JSC “ATI Plant”, Saint-Petersburg, Russia) (Table 1).

Additionally, two types of binder matrices were employed in the composite systems: PF and ER, as detailed in Table 2.

### 2.2. Materials Processing

At the initial stage, the preparation of components was carried out. The selected reinforcing fabrics were pre-cut into rectangular specimens with dimensions of at least 150 × 150 mm. At the second stage, composite samples were fabricated from each type of reinforcing material by stacking multiple layers of fabric of the same type, with intermediate impregnation using either PF or ER. A total of 14 samples were produced: in total, 14 types of composite samples were prepared, 7 using PF and 7 using ER, each corresponding to one of the fabric types. The fabric-to-resin mass ratio was maintained at 65:35%. The number of fabric layers in each sample was adjusted according to the areal density in order to achieve a uniform composite thickness of approximately 4 mm.

The fabric layers were uniformly saturated with the binder to ensure homogeneous polymer distribution and improve interlayer adhesion. After impregnation, the fabric layers were sequentially stacked until the desired thickness of the composite material was achieved.

At the third stage, the prepared specimens underwent compression. The impregnated fabric preforms were placed into a mold under a static load of 100 kg to remove excess resin and prevent porosity formation.

At the final stage, thermal curing of the composite was performed in a muffle furnace. The curing process was carried out at specified temperatures and durations to initiate polymerization of the binder and ensure the formation of a strong material structure.

Specimens impregnated with PF were subjected to a two-step curing cycle. They were heated in the furnace until the temperature reached 120 °C, followed by natural cooling at room temperature for 30 min. The second stage included exposure to 150 °C for one hour, followed by natural cooling until full temperature stabilization.

**Figure 1:**
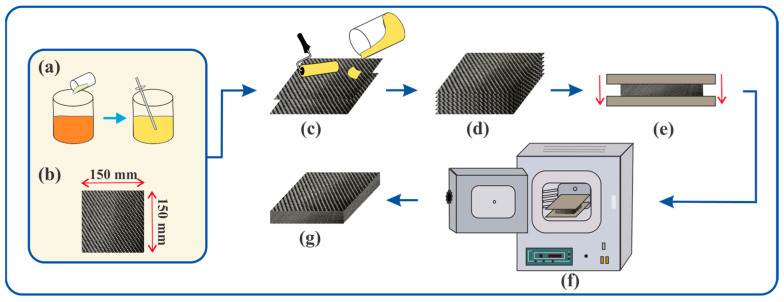
Schematic representation of the composite fabrication process: (**a**) resins: PF and ER; (**b**) reinforcing materials: 7 types of technical fabrics; (**c**) fabric impregnation; (**d**) lamination; (**e**) compression molding; (**f**) thermal curing; (**g**) finished sample.

Specimens impregnated with ER were cured using a stepwise temperature regime: an initial hold at 150 °C for 4 h, followed by heating to 180 °C and holding for 1 h. After the cycle was completed, the furnace was turned off, and the samples were allowed to cool naturally to room temperature. Figure 1 illustrates the complete fabrication process of the composites.

### 2.3. Testing Methods

#### 2.3.1. Tensile and Compressive Testing

The mechanical properties of the composite materials were evaluated via tensile and compressive tests in accordance with the ASTM D3039 and ASTM D6641 standards. Testing was conducted using a universal testing machine RMG-100MG4 (SKB Stroypribor, Moscow, Russia). Composite plate specimens were fabricated and prepared following the guidelines of the respective standards. Each specimen was secured in the grips of the testing machine according to standardized procedures. During tensile testing, load was applied uniformly until specimen failure to determine ultimate tensile strength. Similarly, during compressive testing, the critical load at which local plastic deformation and subsequent failure occurred was recorded.

The RMG-MG4 testing machine applies loads that generate stresses ranging from 0.1 to 1000 MPa, depending on the cross-sectional area of the specimen. The crosshead displacement rate during testing was maintained at 0.2 MPa/s.

All mechanical tests were conducted under standard laboratory conditions (temperature: 23 ± 2 °C, relative humidity: 50 ± 5%). For each composite configuration, a series of five tests was conducted, and average values were used to ensure data reliability.

#### 2.3.2. Hardness Testing

Hardness was measured using a Brinell hardness tester TR5014-01 (LLC “ZIP”, Ivanovo, Russia). The test was conducted by indenting a 10 mm steel ball into the composite surface under a load of 500 kg for 10 s. After load removal, the diameter of the indentation was measured, and Brinell hardness (HB) was calculated according to GOST 9012–2016 [40]. The specimen surfaces were pre-polished to ensure uniform testing conditions and minimize the influence of surface defects. Polishing was performed using sequential abrasive grinding (up to P1200 grit). The traverse speed during indentation was approximately 1 mm/s, and all tests were performed at room temperature. Measurements were taken at three different points for each sample to obtain average hardness values.

#### 2.3.3. Flame Resistance Testing

The fire resistance of the composites was assessed using a propane–oxygen burner, generating flame temperatures of approximately 2000 °C, in accordance with the principles of ISO 2685 and GOST R 52781–2007 [41,42]. Specimens were mounted so that their front face was exposed to direct flame for 30 s, after which the temperature rise on the back surface was recorded in real time using a pyrometer.

Tests were conducted either until the critical temperature threshold was reached or until material failure. The resulting temperature profiles were used to assess the thermal protection efficiency of composites with different reinforcement and matrix configurations. Key evaluation criteria included the following: minimum temperature observed on the rear surface, rate of temperature rise, and the ability of the material to maintain structural integrity under high heat flux conditions.

#### 2.3.4. Thermal Conductivity Measurement

Thermal conductivity was determined using a DRP-II thermal conductivity analyzer. The method was based on measuring heat flux and temperature difference across the sample under a defined thermal regime. The specimen was clamped between a heating and a cooling block, establishing a steady-state or quasi-steady-state thermal field. The analyzer automatically calculated the thermal conductivity coefficient (λ) based on the temperature gradient, specimen thickness, and heat flux data.

Measurements were conducted in accordance with ISO 22007-2:2015 [43]. The experimental setup ensured the accurate determination of λ by maintaining stable and reproducible thermal conditions throughout the test.

#### 2.3.5. Scanning Electron Microscopy (SEM)

Microstructural analysis of the composite specimens was performed using a scanning electron microscope (SEM) in compliance with ISO 16700:2020 [44]. SEM enabled high-resolution imaging of sample surfaces to evaluate fiber morphology, matrix distribution, and the presence of pores, defects, and other structural inhomogeneities.

Images were acquired at accelerating voltages ranging from 5 to 30 kV, depending on material type and specimen characteristics. The resulting micrographs were used to assess impregnation quality, fiber–matrix adhesion, and to quantify porosity and structural imperfections.

## 3. Results and Discussion

### 3.1. Mechanical Properties

Mechanical tests, including hardness, tensile, and compressive strength, were conducted for composite materials reinforced with various fabrics and impregnated with either PF or ER.

**Figure 2:**
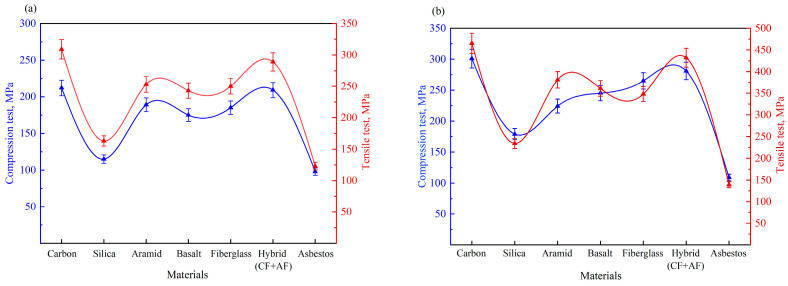
Mechanical properties of the specimens under tension and compression: (**a**) PF; (**b**) ER.

Figure 2a,b show the mechanical performance of composites with PF and ER matrices, respectively. Tensile tests indicated that composites reinforced with ER exhibited superior performance. The highest tensile strength was recorded for carbon fabric + ER at 465 MPa, whereas the corresponding PF-based composite reached only 309 MPa, representing a 50.5% increase. The carbon/aramid hybrid fabric also demonstrated strong performance: 432 MPa (ER) vs. 289 MPa (PF). Aramid-reinforced composites achieved 381 MPa with ER and 253 MPa with PF. Basalt composites reached 361 MPa/243 MPa, and fiberglass composites achieved 348 MPa/250 MPa. Silica-reinforced composites recorded the lowest strength among non-metallic reinforcements: 234 MPa/163 MPa (ER/PF). The asbestos fabric yielded the lowest overall tensile strength: 139 MPa with ER and 123 MPa with PF.

A similar trend was observed in compressive tests. The best performance was again shown by the carbon fabric + ER composite, which reached 301 MPa, 42% higher than its PF counterpart (212 MPa). The hybrid fabric delivered 281 MPa (ER) vs. 209 MPa (PF); basalt: 245 MPa/175 MPa; and aramid: 224 MPa/189 MPa. Fiberglass and silica composites demonstrated moderate strength values: 265 MPa/185 MPa and 179 MPa/115 MPa, respectively. Asbestos composites again showed the lowest strength: 109 MPa (ER) and 98 MPa (PF).

These results confirm that ER-based composites significantly outperform their PF counterparts in terms of both tensile (30–50% average increase) and compressive strength (25–40% increase). The best mechanical performance was observed in composites reinforced with carbon and hybrid fabrics.

**Figure 3:**
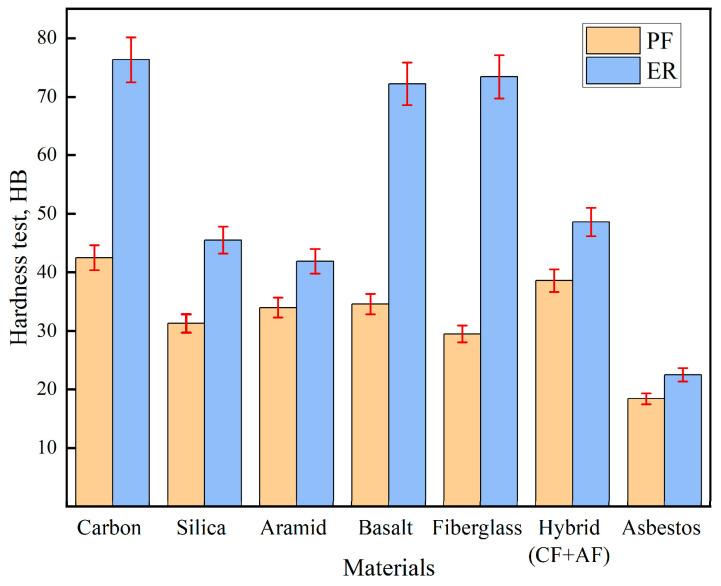
Hardness test results.

Brinell hardness testing revealed that the carbon fabric reinforced with ER exhibited the highest hardness value, 76.3 HB, compared to 42.5 HB with PF (a 79.5% increase). Basalt and fiberglass composites impregnated with ER also demonstrated high hardness values of 72.2 HB and 73.4 HB, respectively. The hybrid carbon/aramid fabric showed a hardness of 48.6 HB with ER and 38.6 HB with PF. The lowest hardness values were recorded for the asbestos fabric: 22.5 HB with ER and 18.4 HB with PF (Figure 3).

The significant difference in mechanical performance (tensile strength, compressive strength, and hardness) can be attributed to the material characteristics of the polymer matrices. ER forms a denser and more rigid cross-linked network upon curing, which enhances load-bearing capacity and resistance to both plastic deformation and indentation. In contrast, PF resins exhibit a more brittle and less compact structure, resulting in lower strength and hardness values. The superior performance of epoxy-based composites is also related to better fiber wetting and stronger interfacial bonding, particularly evident in fiberglass systems. PF matrices, due to their limited penetration ability and lower chemical compatibility with certain fibers, show reduced adhesion and overall mechanical integrity.

### 3.2. Fire Resistance Testing

In the second phase of this study, the thermal conductivity and fire resistance characteristics of the composites were investigated.

**Figure 4:**
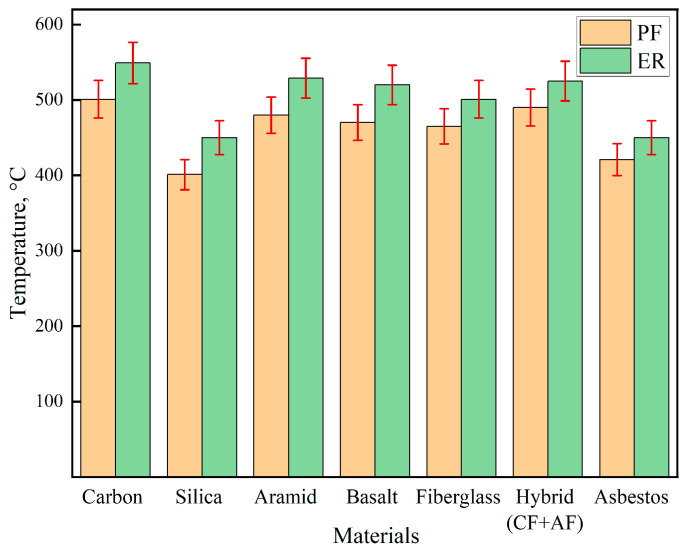
Fire test results.

The comparative analysis of fire resistance, as shown in Figure 4, revealed that the type of reinforcing fabric has a decisive impact on the thermal behavior of the composites under flame exposure. The best performance was achieved with silica fabric, which, in combination with PF, resulted in the lowest back-face temperature of 401 °C. When ER was used instead, the temperature increased to 450 °C. Thus, even with the substitution of the matrix, silica retained its leading position, with only a 12.2% increase in temperature, significantly lower than that observed for other reinforcement types.

Conversely, carbon fabric, despite the high intrinsic thermal resistance of its fibers, exhibited the poorest thermal insulation performance within the composite structure: 501 °C with PF and 549 °C with ER. This corresponds to increases of 22.4% and 37.0%, respectively, compared to the silica + PF configuration. A similar trend was observed for other fabrics: for aramid, the temperature rise between resins was 10.2%, for basalt—10.6%, for fiberglass—7.7%, for the hybrid carbon/aramid fabric—7.1%, and for asbestos—6.9%.

These results indicate that the choice of reinforcing material plays the dominant role in reducing the back-face temperature, while the type of resin serves as a secondary modifying factor. On average, the use of PF led to a 6–12% reduction in temperature compared to ER. The combination of silica fabric and PF demonstrated a pronounced synergistic effect, reducing thermal exposure by 12–37% relative to other configurations, thereby confirming its high effectiveness for use in extreme thermal environments.

Morphological analysis after fire testing was performed using an optical microscope for all 14 composite samples. Optical observations revealed macrostructural changes in the regions exposed to thermal loading. All samples exhibited surface charring and localized discoloration in the flame-affected area. Samples with a PF matrix showed more pronounced cracking and resin shrinkage, whereas epoxy-based materials demonstrated greater structural stability and surface integrity. No significant damage to the reinforcing fibers was observed.

**Figure 5:**
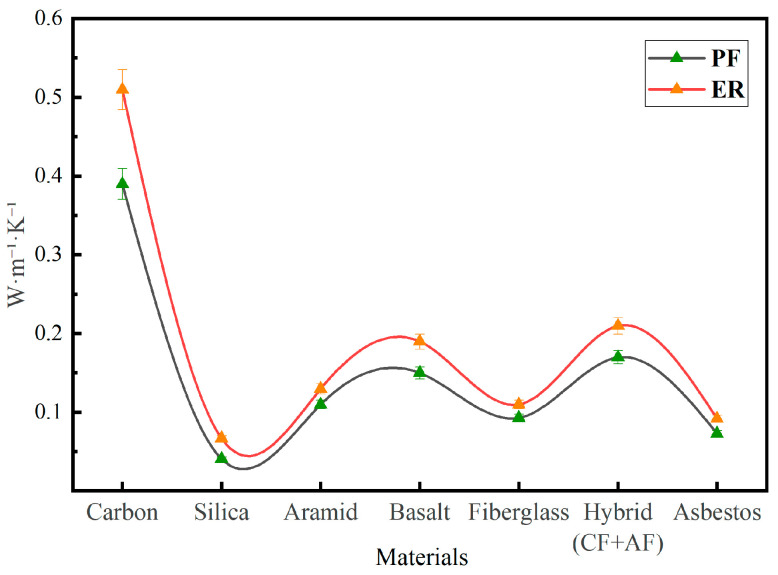
Thermal conductivity test results.

As shown in Figure 5, the highest thermal conductivity is observed in carbon fiber-reinforced composites: ER-based samples reach approximately 0.51 W/m·K, while PF-based ones show around 0.39 W/m·K, a relative increase of about 30%. Silica-based composites demonstrate the lowest thermal conductivity values, from 0.041 W/m·K (PF) to 0.067 W/m·K (ER), indicating an increase of more than 60%. This highlights the sensitivity of low-conductivity fibers to matrix type. Composites reinforced with aramid, basalt, fiberglass, and hybrid (carbon/aramid) fabrics also exhibit increased thermal conductivity when transitioning to ER, with an average growth of 18–27%. For instance, the thermal conductivity of basalt-reinforced composites rises from 0.15 W/m·K to 0.19 W/m·K, while for hybrid ones it increases from 0.17 W/m·K to 0.21 W/m·K.

These results confirm that the use of ER enhances the thermal conductivity of the composites regardless of reinforcement type, and the degree of increase depends on the nature of the fiber and its interaction with the matrix.

This pronounced increase in thermal conductivity for carbon-reinforced composites can be attributed to the intrinsically high thermal conductivity of carbon fibers and the strong interfacial bonding with ER, which reduces thermal resistance at the fiber–matrix interface. In the case of asbestos-reinforced systems, the porous fiber structure allows for deeper resin penetration, and the use of ER helps minimize air voids, resulting in improved heat conduction compared to PF-based composites.

### 3.3. Morphological Analysis

**Figure 6:**
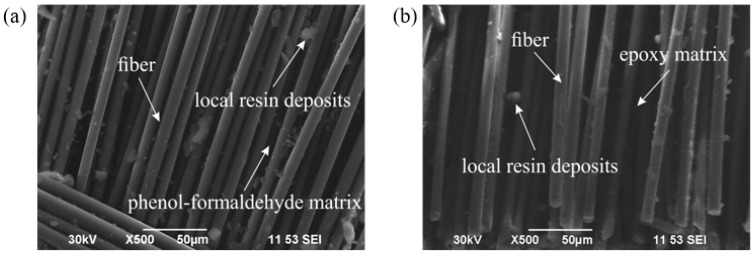
Scanning electron microscope (SEM) images: (**a**) PF; (**b**) ER.

The morphology of composites based on PF and ER matrices, as presented in the SEM images (Figure 6), confirms the fundamental differences between these two types of binders, as previously reported in the literature [22,23,27,28].

PF composite (Figure 6a): The inherent brittleness of PF leads to the formation of microcracks and localized pores at the fiber–matrix interface and within the resin bulk. These defects can negatively impact the mechanical properties under external loads, as they act as stress concentrators and potential initiation sites for material failure. However, when structural integrity is preserved and proper processing parameters are followed, PF-based composites demonstrate excellent thermal resistance, making them well suited for applications where high temperature is a critical factor.

Epoxy composite (Figure 6b): The epoxy matrix, being more viscous and less prone to shrinkage, ensures better fiber impregnation and a lower risk of porosity, clearly visible in SEM images by the absence of significant voids and cracks. The high interfacial adhesion enhances load transfer and increases composite reliability. As a result, these materials exhibit superior mechanical performance, particularly in terms of tensile strength and fatigue resistance, which is essential for structural applications. However, ERs are generally less thermally stable than their phenolic counterparts and require the precise control of curing parameters (temperature, time, and pressure) to achieve optimal performance.

Considering the SEM results in conjunction with the outcomes of fire resistance and mechanical testing, it can be concluded that the silica fabric-based composite offers the best balance between thermal and mechanical performance. This combination of properties is attributed to the high thermal stability of the fibers and their good compatibility with both types of matrices, making this composite system highly promising for applications involving simultaneous thermal and mechanical loading.

## 4. Conclusions

The comprehensive analysis confirmed that the type of reinforcing filler and the nature of the polymer matrix have a decisive influence on the thermomechanical properties of fabric-reinforced composites.

Composites with epoxy matrices demonstrated superior mechanical properties, achieving maximum tensile strength of up to 465 MPa and compressive strength of up to 301 MPa, particularly when reinforced with carbon and hybrid fibers. These elevated values are attributed to the high interfacial adhesion and uniform impregnation of the reinforcement structure provided by the ER.

The lowest back-face temperatures under thermal loading—down to 401—were observed in silica fabric-reinforced composites with a PF matrix. This corresponds to a reduction in thermal exposure of up to 37% compared to other combinations and indicates the effective thermal protection performance of such systems.

The use of PF contributed to a 6–12% decrease in thermal conductivity compared to epoxy-based counterparts. Overall, thermal conductivity values ranged from 0.12 to 0.40 W/m·K, depending on the type of reinforcement.

Morphological analysis revealed stronger interfacial adhesion and greater structural homogeneity in epoxy-based composites, while PF-based systems exhibited higher structural stability under high-temperature loading conditions.

The most balanced combination of mechanical strength and thermal insulation performance was observed in the silica fabric-reinforced composite with a PF matrix, suggesting its strong potential as an effective material for applications involving combined thermal and mechanical stresses.

## Figures and Tables

**Table 1:** Detailed information on the fabric samples.

Fabric Type	Grade	Tensile Strength (MPa)	Young’s Modulus (GPa)	Areal Density, g/m^2^	Temperature Resistance, °C
Carbon fabric	CW180	4000–4900	260	180	up to 2000 (in inert atmosphere)
Silica fabric	KT-11-30K	800–1000	70–80	300	up to 1000
Aramid fabric	Togilen 160	2700–3600	60–145	160	up to 500
Basalt fabric	TBK-100	800–1000	89–100	210	up to 800
Fiberglass fabric	TR-0.7 (100)	1000–1500	70–80	290	up to 600
Hybrid carbon/aramid fabric	C03K15P-195	4000–4500	200–220	195	up to 500–800
Asbestos fabric	AT-16	700–900	50–60	3200	up to 400

**Table 2:** Detailed information on the resin samples.

Characteristic	Phenol-Formaldehyde Resin	Epoxy Resin
Manufacturer	Shandong Shengquan Group Co., Ltd., Jinan, China	JSC “Epital”, Russia
Grade	R-75	Etal-Inject T
Type of Binder	Phenol-formaldehyde, resol-type	Epoxy, thermosetting
Maximum Operating Temperature, °C	up to 250 °C	up to 180–200 °C
Viscosity at 25 °C	2500–3500 mPa·s	400–500 mPa·s
Behavior on Heating	Forms a carbonaceous residue, suitable for carbonization	Forms a heat-resistant residue, not prone to carbonization
Type of Hardener	Does not require hardener (self-curing when heated)	Amino-type, external component

## Data Availability

The original contributions presented in this study are included in the article. Further inquiries can be directed to the corresponding author.

## Abbreviations

The following abbreviations are used in this manuscript:

TPSs	Thermal protection systems
PF	Phenol-formaldehyde resin
ER	Epoxy resin
SRM	Solid rocket motors
SEM	Scanning Electron Microscopy
CF	Carbon fiber
AF	Aramid fiber

## References

[1] Mahjub A., Mazlan N.M., Abdullah M.Z., Azam Q. (2020). Design Optimization of Solid Rocket Propulsion: A Survey of Recent Advancements. J. Spacecr. Rocket..

[2] Uyanna O., Najafi H. (2020). Thermal Protection Systems for Space Vehicles: A Review on Technology Development, Current Challenges and Future Prospects. Acta Astronaut..

[3] Sanoj P., Kandasubramanian B. (2014). Hybrid Carbon-Carbon Ablative Composites for Thermal Protection in Aerospace. J. Compos..

[4] Saba N., Jawaid M. (2018). A Review on Thermomechanical Properties of Polymers and Fibers Reinforced Polymer Composites. J. Ind. Eng. Chem..

[5] Mungiguerra S., Silvestroni L., Savino R., Zoli L., Esser B., Lagos M., Sciti D. (2022). Qualification and Reusability of Long and Short Fibre-Reinforced Ultra-Refractory Composites for Aerospace Thermal Protection Systems. Corros. Sci..

[6] Meiirbekov M., Yermekov A., Nurguzhin M., Kulbekov A. (2025). Identifying the Influence of Winding Angles on the Strength Properties of Carbon Fiber-Reinforced Plastic Tubes. East.-Eur. J. Enterp. Technol..

[7] Meiirbekov M.N., Ismailov M.B. (2020). The Effect of Rubber on the Mechanical Properties of Epoxy and Carbon Fiber (Review). Complex Use Miner. Resour..

[8] Pareek K., Saha P. (2019). Basalt Fiber and Its Composites: An Overview.

[9] Sun G., Tong S., Chen D., Gong Z., Li Q. (2018). Mechanical Properties of Hybrid Composites Reinforced by Carbon and Basalt Fibers. Int. J. Mech. Sci..

[10] Mazraeh-shahi Z.T., Shoushtari A.M., Bahramian A.R. (2015). A New Method for Measuring the Thermal Insulation Properties of Fibrous Silica Aerogel Composite. Procedia Mater. Sci..

[11] Gore P.M., Kandasubramanian B. (2018). Functionalized Aramid Fibers and Composites for Protective Applications: A Review. Ind. Eng. Chem. Res..

[12] Hirai Y., Hamada H., Kim J.-K. (1998). Impact Response of Woven Glass-Fabric Composites-I. Compos. Sci. Technol..

[13] Wang J., Schlagenhauf L., Setyan A. (2017). Transformation of the Released Asbestos, Carbon Fibers and Carbon Nanotubes from Composite Materials and the Changes of Their Potential Health Impacts. J. Nanobiotechnol..

[14] Barato F., Paccagnella E., Franco M., Pavarin D. (2020). Numerical Analyses of Thermal Protection Design in Hybrid Rocket Motors. Proceedings of the AIAA Propulsion and Energy 2020 Forum.

[15] Gale M.P., Alam M.F., Mehta R.S., Luke E.A. (2020). Multiphase Modeling of Solid Rocket Motor Internal Environment. Proceedings of the AIAA Propulsion and Energy 2020 Forum.

[16] Cegła M., Ruliński P., Zmywaczyk J., Koniorczyk P. (2019). Complex Thermal Analysis of Solid Rocket Propellants. AIP Conf. Proc..

[17] Ho D.W.K., Koo J.H., Ezekoye O.A. (2009). Kinetics and Thermophysical Properties of Polymer Nanocomposites for Solid Rocket Motor Insulation. J. Spacecr. Rocket..

[18] Xu Y.H., Hu X., Yang Y.X., Zeng Z.X., Hu C.B. (2014). Dynamic Simulation of Insulation Material Ablation Process in Solid Propellant Rocket Motor. J. Aerosp. Eng..

[19] Park J.K., Kang T.J. (2002). Thermal and Ablative Properties of Low Temperature Carbon Fiber–Phenol Formaldehyde Resin Composites. Carbon.

[20] Haupt R.A., Sellers T. (1994). Characterizations of Phenol-Formaldehyde Resol Resins. Ind. Eng. Chem. Res..

[21] Berdnikova P.V., Zhizhina E.G., Pai Z.P. (2021). Phenol-Formaldehyde Resins: Properties, Fields of Application, and Methods of Synthesis. Catal. Ind..

[22] Pizzi A., Ibeh C.C. (2014). Phenol–Formaldehydes. Handbook of Thermoset Plastics.

[23] Wang X., Xu Q., Zheng Q., Shao Y., Shen J. (2025). Reviews of Fiber-Reinforced Phenolic Resin-Based Thermal Protection Materials for Aircraft. Energies.

[24] Li Y., Shi C., Pan X., Wang Z., Yang L. (2024). Construction of an Interfacial Layer of Aramid Fibers Grafted with Glycidyl POSS Assisted by Heat Treatment and Evaluation of Interfacial Adhesion Properties with Epoxy Resin. ACS Omega.

[25] Koyunbakan M., Uslugil Y., Ekrem M., Eser Ü. (2025). Investigation of the Mechanical Properties of Aramid Fiber-Reinforced Hybrid Nanocomposites with BNNP-Enhanced Epoxy Matrix. Compos. Interfaces.

[26] Jin F.-L., Li X., Park S.-J. (2015). Synthesis and Application of Epoxy Resins: A Review. J. Ind. Eng. Chem..

[27] Mohan P. (2013). A Critical Review: The Modification, Properties, and Applications of Epoxy Resins. Polym. Plast. Technol. Eng..

[28] Ekşı S., Genel K. (2017). Comparison of Mechanical Properties of Unidirectional and Woven Carbon, Glass and Aramid Fiber Reinforced Epoxy Composites. Acta Phys. Pol. A.

[29] Pham H.Q., Marks M.J. (2005). Epoxy Resins. Ullmann’s Encyclopedia of Industrial Chemistry.

[30] Meiirbekov M.N., Ismailov M.B., Kenzhegulov A.K., Mustafa L.M., Tashmukhanbetova I.B. (2024). Study of the Effect of Combined Reinforcement and Modification of Epoxy Resin with Rubbers on the Impact Strength of Carbon Fiber-Reinforced Plastic. Eurasian J. Phys. Funct. Mater..

[31] Dombrovsky L.A., Baillis D. (2010). Thermal Radiation in Disperse Systems: An Engineering Approach.

[32] Natali M., Kenny J.M., Torre L. (2016). Science and Technology of Polymeric Ablative Materials for Thermal Protection Systems and Propulsion Devices: A Review. Prog. Mater. Sci..

[33] German R.M., Messing G.L., Cornwall R.G. (2020). Sintering Technology.

[34] Michaud V., Mortensen A. (2001). Infiltration Processing of Fibre Reinforced Composites: Governing Phenomena. Compos. Part A Appl. Sci. Manuf..

[35] Najmon J.C., Raeisi S., Tovar A. (2019). Review of Additive Manufacturing Technologies and Applications in the Aerospace Industry. Additive Manufacturing for the Aerospace Industry.

[36] Wu H., Koo J.H. (2022). High-Temperature Polymers and Their Composites for Extreme Environments: A Review. Proceedings of the AIAA SCITECH 2022 Forum.

[37] Ma Y., Wang J., Zhao Y., Wei X., Ju L., Chen Y. (2020). A New Vacuum Pressure Infiltration CFRP Method and Preparation Experimental Study of Composite. Polymers.

[38] Servadei F., Zoli L., Galizia P., Melandri C., Sciti D. (2022). Preparation of UHTCMCs by Hybrid Processes Coupling Polymer Infiltration and Pyrolysis with Hot Pressing and Vice Versa. J. Eur. Ceram. Soc..

[39] Martín J., Mijangos C. (2009). Tailored Polymer-Based Nanofibers and Nanotubes by Means of Different Infiltration Methods into Alumina Nanopores. Langmuir.

[40] (2017). Metals. Brinell Hardness Test Method.

[41] (1998). Aircraft—Environmental Test Procedures for Airborne Equipment—Resistance to Fire in Designated Fire Zones.

[42] (2008). Fire-Protective Materials and Products. Test Methods for Ignitability.

[43] (2015). Plastics—Determination of Thermal Conductivity and Thermal Diffusivity—Part 2: Transient Plane Heat Source (Hot Disc) Method.

[44] (2020). Space Systems—Structural Materials and Elements—Test Methods.

